# Management Factors Influence Animal Welfare and the Correlation to Infectious Diseases in Dairy Cows

**DOI:** 10.3390/ani11113321

**Published:** 2021-11-20

**Authors:** Francesca Licitra, Laura Perillo, Francesco Antoci, Giuseppe Piccione, Claudia Giannetto, Rosario Salonia, Elisabetta Giudice, Vincenzo Monteverde, Giuseppe Cascone

**Affiliations:** 1 Istituto Zooprofilattico Sperimentale della Sicilia “A. Mirri”, Via G.Marinuzzi, 3, 90129 Palermo, Italy; francescalicitra15@gmail.com (F.L.); francesco.antoci@izssicilia.it (F.A.); saro.salonia77@gmail.com (R.S.); vincenzo.monteverde@izssicilia.it (V.M.); giuseppe.cascone60@gmail.com (G.C.); 2 Department of Veterinary Science, University of Messina, Polo Universitario dell’Annunziata, 98168 Messina, Italy; lauraperillo77@gmail.com (L.P.); clgiannetto@unime.it (C.G.); elisabetta.giudice@unime.it (E.G.)

**Keywords:** animal welfare, farm animals, dairy cow, intensive housing system, extensive housing system, infectious diseases

## Abstract

**Simple Summary:**

To investigate the relationship between some infectious diseases (*Mycobacterium avium* subsp. paratuberculosis, *Chlamydiophila abortus, Neospora caninum*, bovine viral diarrhea virus, and the bovine herpesvirus) and the dairy farms’ welfare scores, 36 dairy farms were monitored using the Italian National Animal Welfare Reference Center (CreNBA) checklist. Farms and their animals were scored in five different areas, namely: Area A, “Farm management and personnel”; Area B, “Facilities and equipment”; Area C, “Animal-based measures”; Area D, “Inspection of microclimatic environmental conditions and alarm systems”; and Area E, “Biosecurity”. The recorded scores were compared between two farming conditions (access to pasture and indoor housing) and correlated with the serum data. Our results indicated that an accurate application of the checklist could be an instrument to prevent and control the spread of infections in dairy farms.

**Abstract:**

The present study assessed dairy cow welfare through the application of the Italian National Animal Welfare Reference Center (CReNBA) checklist in 36 dairy farms located in Ragusa (Italy) subjected to two different management conditions, housing with free access to pasture (Group 1, farms *n* = 17) and indoor housing (Group 2, farms *n* = 19). Five areas of investigation were considered: Area A, “Farm management and personnel”; Area B, “Facilities and equipment”; Area C, “Animal-based measures”; Area D, “Inspection of microclimatic environmental conditions and alarm systems”; and Area E, “Biosecurity”. Blood samples were collected by coccygeal venipuncture from all animals (4081 cows). The specific antibodies against *Mycobacterium avium* subsp. paratuberculosis, *Chlamydiophila abortus*, *Neospora caninum*, *bovine viral diarrhea virus,* and the *bovine herpesvirus* were assessed by enzyme linked immunosorbent assay (ELISA) serological test. Group 1 (access to pasture) showed a lower value of percentage score recorded in Area A (*p* = 0.02) and E (*p* = 0.01) than Group 2 (indoor housing). *Herpesvirus* (Infectious bovine rhinotracheitis - IBR - detection of gB antibodies/IBR-gB) blood concentrations were higher in the cows housed indoor versus those with access to pasture (*p* = 0.01). Farm management and personnel (score A) was correlated with the level of *bovine viral diarrhea virus* (τ = 0.3754) and bovine-herpesvirus-specific antibodies (IBR-gB) (τ = 0.4159). “Biosecurity” percentage score showed a significant correlation with *Chlamydiophila abortus* (τ = −0.4621) in the cows with access to pasture and IBR-gB (τ = 0.3435) in the cows housed fully indoors. Group 2 showed a significantly reduced level of antibodies against *Neospora caninum*. In conclusion, differences in the welfare assessment score were observed in the “Farm management and personnel” and “Biosecurity” between the two management conditions. It had an effect on the prevalence of herpesvirus, which occurred more in cattle with access to pasture. Therefore, an accurate application of the checklist could be an instrument to prevent and control the spread of infections in farms.

## 1. Introduction

The interest in farm animal welfare assessment is growing [1]. Animal welfare is an essential component of sustainability for the dairy industry [2]. Veterinarians are increasingly being called to be scientific and moral authorities in animal welfare issues [3].

Animal welfare has always been taken into consideration during farming. Farmers have always been attentive to the animals’ conditions to ensure they are healthy and well fed, modifying management practices to improve and optimize the welfare of the herd [1,4]. Throughout the world, dairy cattle are kept in a wide variety of management and housing systems, and in different climate conditions [5]. Animal welfare is a multidisciplinary and dynamic science. Traditionally, the condition of well-being has been seen as the absence of disease or injury. More recently, the issue of animal welfare has focused on the pain or discomfort that animals may have in relation to management practices [4]. Nowadays, animal welfare can be assessed by examining how they interact with the housing conditions, through the use of specific guidance that can be used to evaluate the negative and/or positive impacts of human behavior on animal welfare [6].

Differences in management conditions between farms lead to different welfare levels between herds [7]. Animal welfare is a multidimensional concept; for this reason, the improvement of the level of welfare in a farm by adjusting management or housing factors is complex [8]. The Welfare Quality^®^ protocols for cattle aim to collect information concerning 12 criteria, divided into four essential principles of welfare: good feeding, good housing, good health, and appropriate behavior [7,9]. Each principle must be interpreted on the basis of the special needs of animals of different species exposed to different systems of feeding, housing, and management [10].

In the last half century, animal production systems have undergone a radical transformation that has brought about the concentration of large herds, where the animals are generally kept indoors [11]. Housing cattle indoors year-round reduces labor inputs, facilitates the administration of high-energy diets and increases milk yield without increasing farm size. Cows in indoor housing are also better protected against the adverse effects of extreme climatic conditions and endoparasites. Compared to pasture systems, bedding surfaces can increase the prevalence of integument alterations [12]. 

For several reasons, pasture-based dairy farming is often perceived as advantageous for animal welfare, particularly in comparison to full indoor housing systems [8]. Cows on pasture sometimes experience a lower incidence of diseases such as mastitis and lameness [13]. Moreover, pasture can provide certain welfare benefits—in particular, cows have access to a more natural environment and they can perform behaviors that may be important to them, such as grazing [8]. In nature, health disorders can be infectious or non-infectious. Different conditions or factors relating to the animals (age, parity, lactation stage, breed, immune status), as well as those related to the farming areas (housing, nutrition, climate, management), all contribute to the occurrence of such disorders [14]. 

In 2011, the Istituto Zooprofilattico Sperimentale della Lombardia e dell’Emilia Romagna (IZSLER), with the Italian National Animal Welfare Reference Center (CReNBA), developed a draft welfare assessment protocol and first applied it in dairy farms located in IZSLER’s geographical competence area (Northern Italy). Later on, in 2012–2014, CReNBA’s activities concerning dairy cow welfare assessment covered biosecurity assessment. These were extended to the entire Italian territory, thanks to the coordinated training of several veterinarians from different Italian regions. This protocol is mainly based on findings provided by the European Food and Safety Authority (EFSA) publications, specifically the Welfare Quality^®^ assessment protocol for cattle and the draft regulation under discussion in Strasbourg (“Draft Revised Recommendations concerning Cattle”, revised version No 8, September 2009); minimum requirements provided by the law in force (Legislative Decree 146/2001, transposition of the Council Directive 98/58/EC and Legislative Decree 126/2011, transposition of the Council Directive 2008/119/EC) are also taken into account [14].

The aim of this study was to assess welfare levels through the application of CReNBA’s checklist (File S1) on dairy farms with two different management conditions, and to compare the obtained results with the prevalence of various infectious diseases.

## 2. Materials and Methods

The study was carried out in 36 dairy farms located in the province of Ragusa, Sicily, Italy (36°55′48″ N, 14°44′24″ E and 515 m above sea level), where the climate is warm and temperate. A total of 4081 dairy cows were enrolled in this study. The examined farms had a variable consistency of lactating cows belonging to the following breeds: Italian Friesian, Italian Brown, Red Pied Fleckvieh, and Jersey. The ages ranged from 6 months to 12 years. 

### 2.1. Farms Management

The farms were divided into two groups on the basis of different managements: Group 1, represented by 17 farms, where cows had access to the outdoors; Group 2, represented by 19 farms, where cows were kept indoors full time.

Group 1 cows were kept in a grazing area of about 5–7 hectare (ha), at least 10 hours a day. In these grazing areas, there were herbs typical of the Ragusa plateau, Carob trees (which also acted as shelter), and large pools of water. These areas were bordered by stone walls about one and a half meters high, built with an ancient technique. The cows spent the rest of the day in an area with a barn that served as a refuge (from heat in summer and cold in winter). These barns were generally close to the milking parlor. Artificial insemination was practiced in some farms, while in others natural fertilization with bull was performed. They were fed from May to September with 10 kg of fodder, 15 kg of hay (vetch, oats and barley), and 15 kg of silage (corn or silo grass); while from October to April, they were fed with the same diet, except for silage, as the season allows a lusher pasture. Group 2 cows were kept in a stable with a surface area providing between 6 and 7 m^2^/head or as many usable cubicles according to the number of animals. They were fed with 8 kg of fodder, 14 kg of hay (vetch, oats, and barley), and 23 kg of silage (corn or silo grass). Each farm then had different supplements of 1 to 2 kg (soy, beet, sunflower, cotton, alfalfa). In both groups, water was available ad libitum and the milking routine included pre- and post-dipping.

The average daily milk production was 27 liters in Group 1 and 32 liters in Group 2. In bulk tank milk, the average milk fat composition was 3.68% in Group 1 and 3.60% in Group 2, and the value of milk proteins was 3.50% in Group 1 and 3.40% in Group 2.

The protocol of this study was reviewed and approved in accordance with the standards recommended by the Guide for the Care and Use of Laboratory Animals and the Directive 2010/63/EU. This study did not involve experimental animals. From all animals, 1 blood sample was taken from the caudal vein, while the other observations were done via visual inspection of the animals.

### 2.2. Animal Welfare Assessment

The method we used in this study was based on the analysis of two data groups: the first group represented the hazards occurring as a result of environmental conditions (facilities, equipment, management, and microclimatic conditions), while the second group represented the risks of the relevant adverse effects (health consequences). The hazard assessment was performed using parameters divided into five areas, respectively: Area A—“Farm management and personnel”; Area B—“Facilities and equipment”; Area C—“Animal-based measures”, for carrying out the assessment of the risk and of the consequent negative effects on cattle; Area D—“Inspection of microclimatic environmental conditions and alarm systems”, in the event of serious negative events (e.g., fire); and Area E—“Biosecurity”, to assess the level of prevention against the introduction and/or spread of infectious diseases in the cattle shed.

The checklist that we used consisted of 90 items, each item had three (negative, acceptable, positive) or two (negative and positive) choice options. Not all the inspection items had the same weight in determining the final welfare score; some assessment items had a multiplication or division algorithm that increased or reduced the importance when determining the final welfare score. This protocol was applied by a trained veterinarian on each farm and each checklist, filled in all its parts, was placed online on the appropriate site created at the portal of the CReNBA, which issued a certificate of “Animal Welfare and Biosecurity Assessment” by assigning a score for each of the parameters and an overall score to each farm. The set of these evaluations was subsequently entered into an algorithm that returned a value expressed as a percentage (on a scale from 0 to 100), able to identify the general welfare conditions of the herd. The final calculation of the scores in the various areas and those of the overall welfare and biosecurity was carried out by a specific CReNBA software, available through the website http://benessereruminanti.izsler.it. An overall score was obtained (“not classified”, “acceptable”, “enhanced”, and “excellent”). Each assessment took 2–3 h and was carried out around two hours after milking, between 10:00 and 11:00 a.m. Milking took approximately two hours in each farm. All farms used milking machines. 

### 2.3. Blood Sample Collection

Blood samples from all animals present in the examined farms were collected after animal welfare assessments by coccygeal venipuncture, in the morning, at the same time of day. They were put into vacuum tubes (VacuetteTM, Greiner Bio-One, Rome, Italy) with no anticoagulant additive and centrifuged at 3500 rpm for 10 min. The obtained sera were transferred into plastic tubes. These were analyzed for the detection of specific antibodies, infectious bovine rhinotracheitis-IBR-detection of gB and gE antibodies (IBR-gB and IBR-gE), against *Mycobacterium avium* subsp. paratuberculosis, *Chlamydiophila abortus*, *Neospora caninum*, bovine herpesvirus, and bovine viral diarrhea virus using an indirect enzyme-linked immunosorbent assay (ELISA), as per the manufacturer’s instructions (ID.Vet, Grabels, France). Each serum sample was tested in duplicate and the final results were read by a spectrophotometer, measuring the optical density (OD) at 450 nm. 

### 2.4. Data Analysis

The data collected from the check-list drawn up by the CReNBA and the laboratory assays were entered and stored in a Microsoft Excel spreadsheet, screened for proper coding and errors, and analysis was done. 

The obtained data were expressed as mean ± standard deviation (SD). They were analyzed for normality by Shapiro–Wilk test and for homoscedasticity by Bartlett test. Unpaired *t*-test was applied to assess differences in the studied parameters between the two experimental groups. *p*-value < 0.05 was considered statistically significant. Moreover, Kendall’s tau coefficient (T) was calculated between each area and the amount of specific antibodies recorded for each infection, to assess the relationship between each aspect of breeding (“Farm management and personnel”, “Facilities and equipment”, “Animal-based measures”, “Inspection of microclimatic environmental conditions and alarm systems”, and “Biosecurity”) and the studied infectious diseases. Statistical analysis was performed using the STATISTICA software package (STATISTICA 7 Stat Software Inc., Tulsa, OK, USA).

## 3. Results

The total herd sizes assessed were between 42 to 126 cows per farm, with a total of 1250 cows and a mean value of 73.53 cows/farm, in Group 1 and between 28 to 418 cows per farm, with a total of 2831 cows and a mean value of 149 cows/farm, in Group 2. 

**Figure 1:**
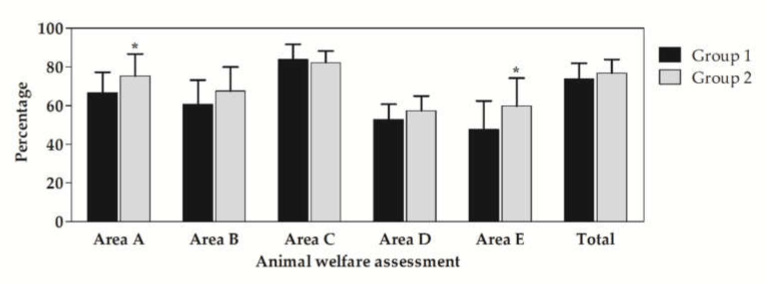
Mean ± standard deviations (±SD) of animal welfare assessment areas (Area A—“Farm management and personnel”; Area B—“Facilities and equipment”; Area C—“Animal-based measures”, for carrying out the assessment of the risk and of the consequent negative effects on cattle; Area D—“Inspection of microclimatic environmental conditions and alarm systems”, in the event of serious negative events (e.g., fire); Area E—“Biosecurity”) in Group 1 (17 farms with an extensive housing system) and Group 2 (19 farms with an intensive housing system). * indicates statistical differences between the two groups (*p* < 0.05).

Figure 1 shows the mean percentages of animal welfare assessment recorded in the two groups in the different checklist areas. In all checklist areas, no differences were observed in the percentage recorded, except in Areas A (*p* = 0.02) and E (*p* = 0.01), for which Group 1 showed a lower value than Group 2. Possible scores range from 0 to 100 and identify the general welfare conditions of the herd, which will then expressed as “not classified”, “acceptable”, “enhanced”, or “excellent”.

**Figure 2:**
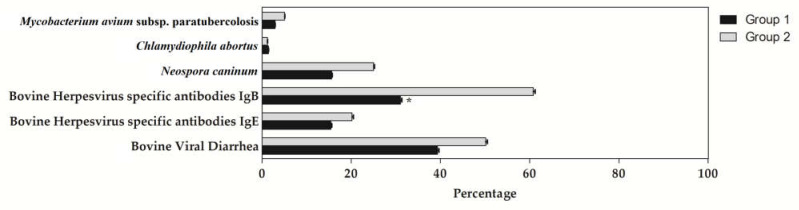
Mean ± standard deviations (±SD) of Mycobacterium avium subsp. paratuberculosis, Chlamydiophila abortus, Neospora caninum, bovine herpesvirus specific antibodies (IBR-gB and IBR-gE), bovine viral diarrhea virus in Group 1 (cows bred with extensive housing system) and Group 2 (cows bred with intensive housing system). * indicates statistical differences between the two groups (*p* < 0.05).

The application of unpaired Student’s *t*-test on the results of ELISA testing for specific antibodies of the diseases investigated showed no differences between the two groups, except for herpesvirus (IBR-gB) (*p* = 0.01), as shown in Figure 2. In group 1, a higher percentage of bovine-herpesvirus-specific antibodies (IBR-gB) than Group 2 was observed (Table 1).

The “Farm management and personal” score was correlated with the bovine-herpesvirus-specific antibodies (IBR-gB) level(τ = 0.41) and bovine viral diarrhea virus (τ = 0.37), while the “Biosecurity” percentage score showed a significant correlation with bovine-herpesvirus-specific antibodies (IBR-gB) (τ = 0.34) in Group 2 and Chlamydiophila abortus (τ = −0.46) in Group 1. Cows housed indoors showed significantly reduced levels of antibodies against Neospora caninum.

## 4. Discussion

Consumers have increased their interest in the security and quality of milk products. Moreover, the interest in the housing and care of dairy cows and their associated products has increased [4,15,16,17]. Management practices and housing systems have been reported to commonly influence animal profitability, health, milk quality, reproductions and well-being, as well as farm productivity [18,19]. 

The CReNBA’s checklist (File S1) represents a functional, reproducible, impartial, and smart instrument based on risk analysis. Using the data collected in each area, it gives a numerical index of animal welfare, providing veterinarians and breeders with the tools to improve farm management and structures, while respecting farm sustainability. For a proper evaluation, it is important to take into account not only the housing facilities, but also the effects of these on the animal. The cows showing discomfort present physical signals that can be observed, interpreted, and evaluated. Such discomfort is frequently linked to pathological conditions (lameness, mastitis, skin alopecia), to abnormal behaviors (fear, aggressiveness), or to alterations in physiological conditions. These situations can be pointed out through animal-based measures in order to detect both health and non-health problems that affect the animals not living in conditions of welfare. The partial result of each area also provides an indication of the burden and importance of each of these on the final calculation of the animal welfare value. Based on the obtained results, the studied animal welfare scores seem to give only limited information about the welfare level of the herd. The evaluation of animal welfare data resulted in different percentages in Areas A and E, with higher values in Group 2 compared to Group 1. These results were in discordance with previous findings that established that intensive housing systems could be associated with many behavioural and welfare problems, in contrast to pasture-based systems, where regular outdoor exercise seems to have positive effects on the health and welfare of dairy cows [11,20,21,22]. Continuously housed systems are perceived to offer less behavioural freedom than pasture-based systems and, as such, are interpreted as offering less welfare [20]. Looking for “normal” cattle behavior, Kilgour [23] identified and reviewed 22 such studies. Cattle have a wide behavioral range, which includes 40 identifiable categories. Grazing was the most common behavior, followed by ruminating and resting, with these three categories accounting for 90% to 95% of an animal’s day. These data revealed most grazing was performed during the daylight, with little grazing at night, while cattle spent more time resting and ruminating at night. Furthermore, there was a diurnal rhythm of behavior, characterized by peaks of grazing activity associated with sunrise and sunset. Few studies have compared dairy cow behavior in pasture vs. continuously housed production systems. Animal welfare is a multi-criteria characteristic [20,24].

The negative correlation between Chlamydiophila abortus and Area E indicated that a better level of biosecurity decreased the spread of this disease, which is in accordance with Anstey et al. [25], who stated that animal husbandry and management systems can potentially influence infection loads in cattle. According to Cascone et al. [26], the negative correlation found between Neospora caninum and Areas C and D in Group 2 showed increasing control in farms with intensive housing systems, reducing the prevalence of infection. The presence of dogs on farm could be a potential risk, increasing the chance of horizontal transmission [27]. Endemic diseases, such as bovine herpesvirus, can be transmitted from a farm to another if protections are not adequate [4,16,28]. Bovine herpesvirus infections were higher in intensive housing systems than extensive housing systems. Positive correlations between Area A and bovine-herpesvirus-specific antibodies (IBR-gB) and bovine viral diarrhea virus, and between Area E and bovine-herpesvirus-specific antibodies (IBR-gB) in Group 2 were also observed. These results are in contrast with Blokhuis et al. [29], who stated that improving animal welfare can enhance the product quality and disease resistance; these effects have direct relevance to food quality and safety. It has also been shown that in the absence of controls, prevalence of bovine herpesvirus is typically high at animal and herd levels [30]. The quality of stockpersonship affects the welfare of animals in the performance of routine tasks such as feeding, cleaning, etc. Assessment of this relationship underlines the importance of stockpersonship in animal welfare. Negative behavior and handling of animals could induce stress and cause injury to animals [31]. Prophylactic measures such as routine diagnosis, reproductive control, and rigorous healthcare protections, including cleaning of facilities, avoiding contact with neighboring herds, acquiring animals with a negative diagnosis, and using an artificial insemination program, should be recommended and implemented in the properties, with the aim of reducing reproductive losses caused by these infections.

## 5. Conclusions

In conclusion, our results showed differences in the “Farm management and personnel” and “Biosecurity” between the two tested management conditions. The two different management conditions had an effect on the herpesvirus prevalence, which was higher when cows had access to pasture compared to when cows were kept indoors full time. The correlation between the different checklist areas tested and the prevalence of different infectious diseases indicated that all aspects of farming were involved in the insurgence of these infectious. Therefore, an accurate application of the checklist could be an instrument for the prevent and control the spread of infections in farms. 

## Figures and Tables

**Table 1:** Mean values and standard deviations (±SD) of Mycobacterium avium subsp. paratuberculosis, Chlamydiophila abortus, Neospora caninum, bovine-herpesvirus-specific antibodies (IBR-gB and IBR-gE), and bovine viral diarrhea virus expressed as percentages obtained from both groups along with statistical results of the unpaired *t*-test.

Infectious disease	Group 1	Group 2	*p*	t	df
*Mycobacterium avium subsp. paratuberculosis*	2.94 ± 0.03	5.04 ± 0.06	0.19	1.31	34
*Chlamydiophila abortus*	1.41 ± 0.02	1.18 ± 0.03	0.80	0.25	34
*Neospora caninum*	15.61 ± 0.11	25.01 ± 0.22	0.12	1.59	34
Bovine herpesvirus specific antibodies (IBR-gB)	31.02 ± 0.30	60.83 ± 0.39	0.01	2.55	34
Bovine herpesvirus specific antibodies (IBR-gE)	15.41 ± 0.21	20.18 ± 0.32	0.60	0.52	34
*Bovine viral diarrhea virus*	39.36 ± 0.33	50.13 ± 0.39	0.38	0.88	34

## Data Availability

Not applicable.

## Supplementary Materials

The following are available online at https://www.mdpi.com/article/10.3390/ani11113321/s1, File S1: Italian National Animal Welfare Reference Center (CReNBA)’s checklist.
