# Supplementary material for: Management Factors Influence Animal Welfare and the Correlation to Infectious Diseases in Dairy Cows
Source: Animals (Basel). 2021 Nov 20;11(11):3321. doi: 10.3390/ani11113321 (PMC8614703; doi:10.3390/ani11113321)
Supplement: Supplementary file 1 [file animals-11-03321-s001.zip › animals-1421649-supplementary.pdf]

NAME OF THE FARM

COMPILER TECHNICIAN

DATE

COMPANY CODE
APA CODE

BREEDS

| | |
|---|---|
| LACTATING COW | |
| DRY COWS | |
| HEIRFER (>6 MONTHS TO $7^{TH}$ PREGNANCY MONTH) | |
| CALVES (<6 MONTHS) | |
| TOTAL CATTLE PRESENT | |

Kg/milk/head/day

Check element **1 Number of stockpersons**

| | |
|---|---|
| → One stockperson (without milking duties) for more than 300 animals<br>→ One stockperson for more than 120 animals (with milking of about 60 animals) | |
| → One stockperson (without milking duties) for more than 200-300 animals<br>→ One stockperson for 80/120 animals (with milking of about 50 animals) | |
| → One stockperson (without milking duties) for less than 200 animals<br>→ One stockperson for less than 80 animals (with milking of about 40 animals) | |

Check element **2 Stockperson training**

**The course must be at least 4 hours long (a half-day or 2 evening slots) and completed in the last 3 years by at least one individual, who may be the owner or an employee**

| | |
|---|---|
| Less than 10 years' experience and no training courses | |
| Minimum 10 years' experience and no training courses | |
| Experience and possession of a qualification or a certificate of attendance of a training course lasting at least ½ day or 2 evening slots in the last 3 years | |

Check element **4 Number of inspection visits of animals**

**Daily inspections are understood as additional to milking activities**

| | |
|---|---|
| 1 inspection/day | |
| 2 inspections/day | |
| >2 inspections/day, written report of the observations (e.g. animals in heat) or computerized recording | |

Check element **5 Typology of equipment for movement**

**It mainly concerns day-to-day movement when sending animals to the milking parlour or changing groups**

| | |
|---|---|
| Using electric goads and sharp instruments | |
| Using only hands or plastic poles | |

Check element **8 Concentrates in the diet (lactating cows)**

| | |
|---|---|
| → More than 60% of DM for diet without corn silage<br>→ More than 50% of DM for diet with corn silage | |
| → Less than 60% of DM for diet without corn silage<br>→ Less than 50% of DM for diet with corn silage | |

Check element **20 Prevention of foot disorders**

| | |
|---|---|
| No trimming programmes and no foot bathing | |
| Yearly trimming programmes and regular foot bathing | |
| Six-monthly trimming programmes and weekly foot bathing | |

Check element **66 Udder health**

| | |
|---|---|
| SCC geometric average > 400.000 cell/ml | |
| SCC geometric average between 300.000 and 400.000 cell/ml | |
| SCC geometric average < 300.000 cell/ml | |

Check element **68 Annual mortality in adult cows**

**Determine the no. of adult cows naturally dead in the barn / euthanatized / urgent slaughtered in the last 12 months**

| | |
|---|---|
| > 5% | |
| Between 2% and 5% | |
| < 2% | |

| Check element | 69 | Annual mortality in calves | |
|---|---|---|---|
| **Determine the no. of calves dead from day 2 to day 30 of life, excluding stillbirths, during the last 12 months** | | | |
| > 10% | | | |
| Between 4% and 10% | | | |
| < 4% | | | |

| Check element | 79 | Purchasing and/or moving animals outside the farm | |
|---|---|---|---|
| Purchasing and moving animals (fairs/shows/markets) as a routine | | | |
| Purchasing only in an emergency (last 3 years) and not moving animals | | | |
| Neither purchasing nor moving animals for over 3 years | | | |

| Check element | 82 | Control and prevention of major infectious diseases | |
|---|---|---|---|
| **Based on knowledge of the major diseases and implementation of relevant control plans (IBR/BVD/PTBC). To get the maximum score, a farmer must know all 3 diseases and implement control plans on at least two of them** | | | |
| No knowledge | | | |
| Partial knowledge and undefined control plans | | | |
| Knowledge of infection status and operational prevention plans in place | | | |

| Check element | 85 | Control and analysis of water sources | |
|---|---|---|---|
| **The analysis is valid even if only microbiological** | | | |
| Lack of water analysis | | | |
| Yearly analysis/municipal aqueduct water | | | |

| Check element | 86 | Source of drinking water | |
|---|---|---|---|
| From one source only (well or aqueduct) without reserve tank | | | |
| From aqueduct or well with a large tank sufficient for some hours | | | |
| From more than one source (aqueduct and well or two wells) | | | |

| Check element | 88 | Ventilation system alarm | |
|---|---|---|---|
| No | | | |
| Yes | | | |

| Check element | 89 | Fire alarm | |
|---|---|---|---|
| No | | | |
| Yes | | | |

| Check element | 90 | Backup power generator | |
|---|---|---|---|
| Lack of backup power generator | | | |
| Presence of backup power generator /or lack of backup power generator but herd has fewer than 10 lactating cows (milkable by hand) | | | |

| Check element | 3 | Group management | |
|---|---|---|---|
| **Additional groups are contemplated only among dry cows or lactating cows** | | | |
| No groups or only heifers/cows | | | |
| No. of groups corresponding to the number of assessment categories (heifers, dry cows, lactating cows) | | | |
| Several groups (transition cows, first-calf heifers, etc.) of lactating or dry animals; or 3 clearly separate main groups in herds with fewer than 50 animals overall gruppi base ben distinti | | | |

| Check element | 7 | Diet | |
|---|---|---|---|
| **The specific diet must be written or attached to the weighing machine. There must be documentary evidence of traceability** | | | |
| Empirical ration without calculating nutritional requirements | | | |
| Specific ration for each main group (heifers – dry cows – lactating cows) | | | |
| Calculated ration by a nutritionist, with a traceability system and correct storage methods | | | |

| | 18 | Calving area management | |
|---|---|---|---|
| **The calving pen may be individual or multiple. The management of the time of movement of the cows and the management of the hygiene of the litter are evaluated.** | | | |
| No calving pen or displacement at wrong times and unsuitable litter hygienic conditions | | | |
| Presence of calving pen, respect of moving time and acceptable litter hygiene | | | |
| Presence of calving pen with wide spaces (>7 m²/head) cleaned on deep litter and respect of moving time | | | |

| Check element | 22 | Management of the milking operations and udder hygiene | |
|---|---|---|---|
| **Milking is "inadequate" when performed incorrectly and when clusters are prepared and attached in the wrong order, leading to overmilking in the majority of cows milked** | | | |
| Milking unit used incorrectly and clusters incorrectly attached and detached (lack of hygiene or failure to observe oxytocin lag times) | | | |
| Correct management of milking operations and equipment (adequate cleaning and observance of lag times) | | | |
| Adequate cleansing, observance of lag times and use of pre/post-dipping with a spray or clean dip cups | | | |

| Check element | 47 | Hospital pen | |
|---|---|---|---|
| **Cubicles are not recommended in a hospital pen. Only an intermediate rating can therefore be assigned where there is a hospital pen with cubicles** | | | |
| Lack of hospital pen | | | |
| Presence of hospital pen, with cubicles or acceptable managed deep litter | | | |
| Presence of hospital pen, with optimally managed deep litter and the possibility to milk cows using a mobile milking unit, or hospital pen located close to the milking parlour | | | |

| Check element | 48 | Waiting and milking parlours | |
|---|---|---|---|
| **Assessment of the waiting area concerns how easy it is to access the milking parlour, the surface area/animal and maximum waiting time for the largest cattle group** | | | |
| Small waiting area (<1.6m²/head) or difficult to enter the milking parlour or waiting >90 min | | | |
| Adequate waiting area (1.6-1.8 m²/head), normal entry with waiting times between 90 and 60 min | | | |
| Wide waiting area (> 1.8 m²/head) with waiting times <60 min. or robotic milking system | | | |

| Check element | 49 | Milking parlour maintenance | |
|---|---|---|---|
| Basic parameters not known (vacuum level – how often sleeves are changed) and there is no service schedule | | | |
| Regular general checks are carried out and specialistic servicing is only performed in case of breakdown, or absence of documentation certifying careful regular servicing | | | |
| There is a servicing program and there are records taken by stockpersons | | | |

| Check element | 50 | Temperature and humidity | |
|---|---|---|---|
| **An open barn is considered when there is no walls on any of the 4 sides or when there is only one closed side, without this limiting good ventilation of the barn** | | | |
| Inadequate temperature and humidity, closed buildings or semi-open barns without adequate air circulation | | | |
| Adequate temperature and humidity with natural ventilation (open barn) / air circulation systems without THI detection control units | | | |
| Presence in the most important housing areas (dry, calving area and lactation area) of cooling systems with THI detection control units/ pasture | | | |

| Check element | 52 | Lighting | |
|---|---|---|---|
| No artificial lighting | | | |
| Artificial lighting available | | | |
| Correct artificial lighting (at least 40 lux) even at night | | | |

| Check element | 65 | Lameness | |
|---|---|---|---|
| **No. of lame lactating and dry cows (including those in the hospital pen) out of actual no. of cows** | | | |
| >8% lame cows | | | |
| Between 8 and 4% lame cows | | | |
| <4% lame cows | | | |

| Check element | 67 | No. of treatments for clinical mastitis in a year | |
|---|---|---|---|
| **Number of antibiotic treatments for clinical mastitis in the past 12 months versus the number of lactating cows at the time of the visit. The response can be verified through the drug registry** | | | |
| No. of mastitis treatments more than 80% of lactating cows number or data unavailable | | | |
| No. of mastitis treatments between 40 and 80% of lactating cows number | | | |
| No. of mastitis treatments less than 40% of lactating cows number | | | |

| Check element | 70 | Mutilations | |
|---|---|---|---|
| Mutilations not authorized by law and/or cows with and without horns mixed together | | | |
| Mutilations authorized by law | | | |
| No mutilation | | | |

| Check element | 71 | General biosecurity procedures – Rodents and insects control |
|---|---|---|
| **An interim judgment is assigned if there is a definite and comprehensive plan for combating rodents and insects (flies)** | | |
| No procedures (to fight undesired animals) | | |
| Extant procedures are approximate and not formalized (no written plans) | | |
| Comprehensive written procedures in place or available from a biosecurity manual | | |

| Check element | 72 | Other animal species inside the farm |
|---|---|---|
| **Are there other species inside the farm area such as farm animals (pigs, sheep and goats), and/or beef cattle and/or domestic animals (dogs, cats, poultry, columbiformes, etc.)?** | | |
| Yes | | |
| No | | |

| Check element | 73 | General biosecurity procedures - Strangers entering the premises |
|---|---|---|
| **An interim judgment is assigned if there is a registering procedure and there are clear and respected warning signs forbidding entry to strangers** | | |
| No procedures (men and vehicles entering the premises) | | |
| Extant procedures are approximate and not formalized (no written plans) | | |
| Comprehensive written procedures in place or available from a biosecurity manual | | |

| Check element | 74 | Handling visitors |
|---|---|---|
| **Visitors include veterinarians, farm consultants in general, etc.** | | |
| No precaution regarding clothing | | |
| All “visitors” wear disposable boot covers | | |
| All “visitors” must go to through a dressing area and are required to wear disposable gowns and boot covers provided by the farm or clothing that remains on-site (e.g., the farm’s veterinarian) | | |

| Check element | 75 | Disinfecting vehicles at the entrance to the farm |
|---|---|---|
| No controls | | |
| There are protective sanitizing procedures but they are not employed routinely (only for emergencies) | | |
| There are protective sanitizing procedures and they are always used | | |

| Check element | 76 | Possibility of contact between outside vehicles and farm animals |
|---|---|---|
| **Do feed and milk trucks transit through areas that allow direct or indirect contact (< 20 m) with the animals?** | | |
| Yes | | |
| No | | |

| Check element | 77 | Collecting animal carcasses |
|---|---|---|
| **Do trucks for the collection of dead animals transit through areas that allow direct or indirect contact (< 20 m) with the live** | | |
| Yes | | |
| No, the vehicle is stopped at the farm’s border, where carcasses have been previously collected | | |

| Elemento di verifica | 78 | Loading live animals (e.g., for sale) |
|---|---|---|
| Animals are loaded inside farm premises (>20 m) | | |
| Animals are loaded outside farm premises (<20 m) | | |

| Check element | 80 | Quarantine |
|---|---|---|
| No quarantine of any kind | | |
| Yes, partial: quarantine performed in an empirical and not formalized way (dedicated area adjacent to the other animals/things done in a short time/no biological test) | | |
| Yes, correct: no animals purchased for years, or compliance with an adequate quarantine in terms of time and facilities, complete with biological tests on the purchased animals | | |

| Check element | 81 | Mastitis control and prevention |
|---|---|---|
| **The farmer must be in possession of a recent analytical microbiological result (last 12 months) from bulk milk or individual milk samples, indicating the risk of mastitis** | | |
| No analysis | | |
| Only bulk analysis for contagious mastitis monitoring | | |
| Analysis on problematic individuals and consequent eradication and control plans | | |

Check element **83 Health condition pertaining to IBR**

**What is the biosecurity level concerning the prevention of IBR in the farm? The monitoring needs to be supported by diagnostic tests carried out in the last 12 months**

| | |
|---|---|
| **High-risk farm**: the livestock are positive or the farmer does not know the situation of his livestock, or during the last 12 months the farmer introduced animals of unknown health status without carrying out any serological tests | |
| **Controlled-risk farm**: the livestock are positive, but a vaccination using a marker vaccine is carried out and, at a serological control carried out in the last 12 months, all of the animals under 36 months tested negative; no animals have been introduced in the last 12 months or only seronegative animals have been introduced and their negativity has been controlled whilst in quarantine | |
| **Low-risk farm**: the livestock give a negative reaction to a serological test carried out in the last 12 months on all the animals or on a statistically significant animal sample (IC = 95%; PA ≥ 5%); no animals have been introduced in the last 12 months or only seronegative animals have been introduced and their negativity has been controlled whilst in quarantine/or farm officially free | |

Check element **84 Health condition pertaining to Paratuberculosis**

**What is the biosecurity level concerning the prevention of ParaTBC in the farm? The monitoring needs to be supported by diagnostic tests carried out in the last 12 months**

| | |
|---|---|
| The farmer does not know the situation of his own farm, or the farmer introduced animals of unknown health status during the last 12 months or the farm has a > 5% infection percentage | |
| The farm tested negative (<5%) to a serological test carried out on all the animals according to the S1-type protocolA and no animals have been introduced in the last 12 months (or only animals coming from farms with equal health level have been introduced) | |
| The farm gives a negative reaction to at least a serological test carried out according to the S2-type protocolB and no animals have been introduced in the last 12 months (or only animals coming from farms with equal health level have been introduced) | |

Check element **12 Cleanliness of water troughs (heifers)**

**See reference photo. The water in the dirty trough is not clear; an intermediate judgment is given if there is a small amount of feed spoilage on the surface or on the bottom of the trough but the water is clear**

| | |
|---|---|
| There is dirt on the surface and walls of the troughs | |
| There is feed either on the surface or on the bottom. The water must be clear | |
| No dirt, the troughs are clean and the water clear | |

Check element **15 Cleanliness of floors (aisles and passageways) (heifers)**

**Examine not only the cleanliness of the floors but also that of the lower part of the limb. Take into consideration the conditions in which the majority of the individuals live**

| | |
|---|---|
| Prevalence of dirty areas in almost all groups | |
| Areas sufficiently clean in almost all groups | |
| All areas properly managed, clean and dry in all groups | |

Check element **19 Litter management (deep litter or cubicles) (heifers)**

**Examine litter hygiene (not the type of material used) and take account of restoration and replacement management. In the case of groups belonging to the same category, consider the living conditions of the majority of subjects**

| | |
|---|---|
| Dirty litter | |
| Litter not very clean and managed sufficiently | |
| Clean litter, topped up or attended to daily and renewed regularly | |

Check element **28 Heifer housing**

**Assessment should be made on indoor deep litter used for lying down. The surface areas of alleys, exercise areas or areas for accessing the feeding trough are excluded**

| | |
|---|---|
| Less than 3.5-4 $m^2$/head or insufficient cubicles | |
| 3.5-4 $m^2$/head or as many cubicles as heifers | |
| More than 4 $m^2$/head or surplus cubicles | |

Check element **32 Litter material (heifers)**

**Do not contemplate the hygiene conditions of the litter material, but just which type of material is used and the damage that it could cause (e.g. lacking, inadequate, mouldy, abrasive, etc.)**

| | |
|---|---|
| No litter material whatsoever | |
| Presence of mats or sufficient material | |
| Presence of appropriate material | |

Check element **35 Flooring (heifers)**

**The animals must be able to move about, even quickly, without slipping**.

| | |
|---|---|
| Inadequate, smooth and slippery slatted or solid concrete flooring | |
| Adequate slatted or solid concrete flooring over at least 50% of the surface area | |
| Adequate slatted or solid concrete flooring on the overall surface area | |

Check element 38 **Number of places available at the feeding trough (heifers)**

**The places must be at least 68 cm each (50 cm for heifers), therefore, if they are lacking, a cow should have 68 cm space at the feeding trough (50 cm for heifers)**

| | |
|---|---|
| → Number of feeding places <100% of the animals in case of conventional feeding<br>→ Number of feeding places <70% of the animals in case of total mixed ration | |
| → Number of feeding places equal to 100% of the animals in case of conventional feeding<br>→ Number of feeding places >70% of the animals in case of total mixed ration | |
| Feeding troughs on two different barn areas and number of feeding places greater than the number of animals or possibility (even for a period of 60 days/year) of access to pasture | |

Check element 42 **Size of drinking troughs (heifers)**

**There must be at least one drinking bowl every 15 heifers or 5 cm/head in case of water troughs**

| | |
|---|---|
| Number or size less than the minimum required | |
| Number and size equal to the minimum required | |
| Number and size higher than expected and with well diversified locations | |

Check element 43 **Litter for calves younger than 2 weeks**

**It is enough to find the presence of a single calf, younger than 2 weeks, housed in conditions not conform to the law requirement (lack of litter or litter harmful for the calf) to assign the pejorative judgment**

| | |
|---|---|
| Lack of litter | |
| Litter used in a way that allows the calf to lie down fully on suitable material | |

Check element 44 **Surface area for calves housed in individual pens**

**Legal dimensions are a length of 130 cm and a width of 80 cm. It is enough to find the presence of a single calf housed in conditions not conform to the law requirement to assign the pejorative judgment**

| | |
|---|---|
| Dimensions smaller than legal limits or presence of calves which are tethered at all times | |
| Dimensions in compliance with legal limits (= or >10%) | |
| Dimensions greater than legal limits | |

Check element 45 **Possibility of contact with other calves**

**The front of the adjacent feeding trough is not to be considered as contact. It is enough to find the presence of a single calf housed in conditions not conform to the law requirement to assign the pejorative judgment**

| | |
|---|---|
| No | |
| Yes | |

Check element 46 **Surface area for calves housed in multiple pens**

**Assess calves under 6 months of age in multiple pens. The surface area is to be understood as the total surface area of the pen(<150 kg l.w. = 1.5 $m^2$/head; between 150 and 220 kg l.w. = 1.7 m2 /animal; > 220kg l.w. = 1.8 $m^2$/head).It is enough to find the presence of a single calf housed in conditions not conform to the law requirement to assign the pejorative judgment**

| | |
|---|---|
| Dimensions below the legal limits or one or more calves over 8 weeks in a single box / or one or more calves constantly tied | |
| Dimensions compliant or slightly higher (10%) than legal limits | |
| Dimensions exceeding the legal limits (at least more than 10%) and / or automatic milk distribution | |

Check element 55 **Flight from humans test (heifers)**

**The final score is obtained by calculating the average of the scores given to each animal, rounding off to the nearest whole number**

| | |
|---|---|
| Difficulty approaching and presence of stereotypies | |
| Curious animals that can be approached and do not show stereotypies | |
| Curious animals that can be approached and let themselves be touched | |

Check element 58 **State of nutrition (heifers)**

**BCS values lower than 2 and greater than 4.25 are beyond acceptable limits**

| | |
|---|---|
| >10% of the animals with BCS beyond set limits | |
| <10% of the animals with BCS beyond set limits | |
| <5% of the animals with BCS beyond set limits | |

Check element **61** **Cleanliness of animals (heifers)**

**For each animal consider the observation of only one side**

| | |
|---|---|
| Over 20% of the animals are dirty | |
| Between 10% and 20% of the animals are dirty only in some groups | |
| Less than 10% of the animals are dirty in all the groups | |

Check element **64** **Skin lesions (heifers)**

**Lesions are assessed by observing the cows starting from the head and moving toward the tail: sternum, shoulder, forelimb, pelvis, thigh, hind limb (lateral and medial) and udder**

| | |
|---|---|
| Over 30% of the animals have minor skin lesions on hocks, tuberosities and soft tissues | |
| Between 15% and 30% of the animals have minor skin lesions on hocks, tuberosities and soft tissues | |
| Less than 15% of the animals have minor skin lesions on hocks, tuberosities and soft tissues | |

Check element **11** **Cleanliness of water troughs (dry cows)**

**The water in the dirty trough is not clear; an intermediate judgment is given if there is a small amount of feed spoilage on the surface or on the bottom of the trough but the water is clear**

| | |
|---|---|
| There is dirt on the surface and walls of the troughs | |
| There is feed either on the surface or on the bottom. The water must be clear | |
| No dirt, the troughs are clean and the water clear | |

Check element **14** **Cleanliness of floors: (aisles and passageways) (dry cows)**

**Examine not only the cleanliness of the floors but also that of the lower part of the limb. Take into consideration the conditions in which the majority of the individuals live**

| | |
|---|---|
| Prevalence of dirty areas in almost all groups | |
| Areas sufficiently clean in almost all groups | |
| All areas properly managed, clean and dry in all groups | |

Check element **17 Litter management (deep litter or cubicles) (dry cows)**

**Examine litter hygiene (not the type of material used) and take account of restoration and replacement management. In the case of groups belonging to the same category, consider the living conditions of the majority of subjects**

| | |
|---|---|
| Dirty litter | |
| Litter not very clean and managed sufficiently | |
| Clean litter, topped up or attended to daily and renewed regularly | |

Check element **26** **Surface area provided for lying down (dry cows)**

**The assessment should be performed on indoor deep litter used for lying down. The surface areas of aisles, exercise areas or areas giving access to the feeding trough are excluded**

| | |
|---|---|
| Less than 6 $m^2$/head or no. of usable cubicles <90% of the animals | |
| Between 6 and 7 $m^2$/head or as many usable cubicles as animals | |
| More than 7 $m^2$/head or no. of usable cubicles > 110 % of the animals | |

Check element **27** **Surface area provided for lying down (cows at calving)**

**The assessment should be performed on indoor deep litter used for lying down. If the farm has less than 5/6 dry subjects and does not have the pre-calving group, the evaluations of item 26 (dry) must be resumed**

| | |
|---|---|
| Less than 6 $m^2$/head or no. of usable cubicles <90% of the animals | |
| Between 6 and 7 $m^2$/head or as many usable cubicles as animals | |
| More than 7 $m^2$/head or no. of usable cubicles > 110 % of the animals | |

Check element **31** **Litter material (dry cows)**

**Do not contemplate the hygiene conditions of the litter material, but just which type of material is used and the damage that it could cause (e.g. lacking, inadequate, mouldy, abrasive, etc.)**

| | |
|---|---|
| No litter material whatsoever | |
| Presence of mats or sufficient material | |
| Presence of appropriate material | |

Check element **34** **Flooring: lactating cows (dry cows)**

**The animals must be able to move about, even quickly, without slipping**

| | |
|---|---|
| Inadequate, smooth and slippery slatted or solid concrete flooring | |
| Adequate slatted or solid concrete flooring over at least 50% of the surface area | |
| Adequate slatted or solid concrete flooring on the overall surface area | |

Check element **37** **Number of places available at the feeding trough (dry cows)**

**The places must be at least 68 cm each (50 cm for heifers), therefore, if they are lacking, a cow should have 68 cm space at the feeding trough (50 cm for heifers)**

| | |
|---|---|
| → Number of feeding places <100% of the animals in case of conventional feeding<br>→ Number of feeding places <70% of the animals in case of total mixed ration | |
| → Number of feeding places equal to 100% of the animals in case of conventional feeding<br>→ Number of feeding places >70% of the animals in case of total mixed ration | |
| Feeding troughs on two different barn areas and number of feeding places greater than the number of animals accesso al<br>or possibility (even for a period of 60 days/year) of access to pasture | |

Check element **41** **Size of drinking troughs (dry cows)**

**There must be at least one drinking bowl every 10 cows or 6-7 cm/head in case of water troughs**

| | |
|---|---|
| Number or size less than the minimum required | |
| Number and size equal to the minimum required | |
| Number and size higher than expected and with well diversified locations | |

Check element **54** **Flight from humans test (dry cows)**

**The final score is obtained by calculating the average of the scores given to each animal, rounding off to the nearest whole number**

| | |
|---|---|
| Difficulty approaching and presence of stereotypies | |
| Curious animals that can be approached and do not show stereotypies | |
| Curious animals that can be approached and let themselves be touched | |

Check element **57** **State of nutrition (dry cows)**

**BCS values lower than 2 and greater than 4.25 are beyond acceptable limits**

| | |
|---|---|
| >10% of the animals with BCS beyond set limits | |
| <10% of the animals with BCS beyond set limits | |
| <5% of the animals with BCS beyond set limits | |

Check element **60** **Cleanliness of animals (dry cows)**

**For each animal consider the observation of only one side**

| | |
|---|---|
| Over 20% of the animals are dirty | |
| Between 10% and 20% of the animals are dirty only in some groups | |
| Less than 10% of the animals are dirty in all the groups | |

Check element **63** **Skin lesions (dry cows)**

**Lesions are assessed by observing the cows starting from the head and moving toward the tail: sternum, shoulder, forelimb, pelvis, thigh, hind limb (lateral and medial) and udder**

| | |
|---|---|
| Over 30% of the animals have minor skin lesions on hocks, tuberosities and soft tissues | |
| Between 15% and 30% of the animals have minor skin lesions on hocks, tuberosities and soft tissues | |
| Less than 15% of the animals have minor skin lesions on hocks, tuberosities and soft tissues | |

Check element **6** **Moving cows at milking**

**In order to give the best judgment, none of the problems described must be present for at least the majority of the groups milked**

| | |
|---|---|
| Impediments to the access of the milking parlour or inadequate stalls or slippery floor or tricky exit with curves (180°) or obstacles | |
| Milking parlour easily accessed and exited | |

Check element **9** **Water availability (all groups)**

| | |
|---|---|
| Absence of drinking water for one or more animals | |
| Presence of functioning drinkers in all the groups | |
| Water given (in addition to the milk and at least once a day) even to calves in individual pens. Where calves are in mixed conditions, assessment is performed on the situation in which most of them are | |

Check element **10** **Cleanliness of water troughs (lactating cows)**

**The water in the dirty trough is not clear; an intermediate judgment is given if there is a small amount of feed spoilage on the surface or on the bottom of the trough but the water is clear**

| | |
|---|---|
| There is dirt on the surface and walls of the troughs | |
| There is feed either on the surface or on the bottom. The water must be clear | |
| No dirt, the troughs are clean and the water clear | |

Check element **13** **Cleanliness of floors: (aisles and passageways) (lactating cows)**

**Examine not only the cleanliness of the floors but also that of the lower part of the limb. Take into consideration the conditions in which the majority of the individuals live**

| | |
|---|---|
| Prevalence of dirty areas in almostall groups | |
| Areas sufficiently clean in almost all groups | |
| All areas properly managed, clean and dry in all groups | |

Check element **16** **Litter management (deep litter or cubicles) (lactating cows)**

**Examine litter hygiene (not the type of material used) and take account of restoration and replacement management. In the case of groups belonging to the same category, consider the living conditions of the majority of subjects**

| | |
|---|---|
| Dirty litter | |
| Litter not very clean and managed sufficiently | |
| Clean litter, topped up or attended to daily and renewed regularly | |

Check element **21** **Hygiene in the milking parlour**

**Assess the general hygiene of the parlour and not its level of technology**

| | |
|---|---|
| Presence of faeces on the milking clusters and on the walls | |
| Adequate cleaning of clusters, but faeces on the floors and walls | |
| No faeces present and good general hygiene | |

Check element **24** **Type of housing system of animals over six months**

**The observation should be performed on all animals, over six months**

| | |
|---|---|
| Even just one animal group is tethered | |
| All animal groups are loose housed | |
| Adult dairy cows are loose housed and can have access to an exercise area equal to 4-5 square meters / head and / or a pasture adjacent to the buildings or provided with adequate shelter | |

Check element **25** **Surface area provided for lying down (lactating cows)**

**The assessment should be performed on indoor deep litter used for lying down. The surface areas of aisles, exercise areas or areas giving access to the feeding trough are excluded**

| | |
|---|---|
| Less than 6 m /head or no. of usable cubicles <90% of the animals | |
| Between 6 and 7 m /head or as many usable cubicles as animals | |
| More than 7 m /head or no. of usable cubicles >110% of the animals | |

Check element **29** **Suitability of the resting area (lactating cows)**

**The number of animals lying down should be counted at least 2 hours after operations such as feeding or milking. In case of an uncertain number, assess the activity and the way in which subjects are standing, or the cubicle design**

| | |
|---|---|
| Poorly exploited cubicles or deep litter (<50%) / or cubicles with incorrect dimensions | |
| Partial use of cubicles or deep litter (between 50 and 70%) / or cubicles with sufficient dimensions | |
| Complete and uniform use of deep litter and/or cubicle resting spaces, or with ideal dimensions | |

Check element **30** **Litter material (lactating cows)**

**Do not contemplate the hygiene conditions of the litter material, but just which type of material is used and the damage that it could cause (e.g. lacking, inadequate, mouldy, abrasive, etc.)**

| | |
|---|---|
| No litter material whatsoever | |
| Presence of mats or sufficient material | |
| Presence of appropriate material | |

Check element **33** **Flooring (lactating cows)**

**The animals must be able to move about, even quickly, without slipping**

| | |
|---|---|
| Inadequate, smooth and slippery slatted or solid concrete flooring | |
| Adequate slatted or solid concrete flooring over at least 50% of the surface area | |
| Adequate slatted or solid concrete flooring on the overall surface area | |

Check element **36** **Number of places available at the feeding trough (lactating cows)**

**The places must be at least 68 cm each (50 cm for heifers), therefore, if they are lacking, a cow should have 68 cm space at the feeding trough (50 cm for heifers)**

| | |
|---|---|
| → Number of feeding places <100% of the animals in case of conventional feeding<br>→ Number of feeding places <70% of the animals in case of total mixed ration | |
| → Number of feeding places equal to 100% of the animals in case of conventional feeding<br>→ Number of feeding places >70% of the animals in case of total mixed ration | |
| Feeding troughs on two different barn areas and number of feeding places greater than the number of animals accesso al<br>or possibility (even for a period of 60 days/year) of access to pasture | |

Check element **39** **Size and characteristics of the single post in the rack (lactating cows)**

**Evaluate the characteristics of the access to the feeding place of the individual subject, considering the rack type and the complete size of the single access**

| | |
|---|---|
| Space in the feeding place for animals less than 68 linear cm / other obvious access difficulties | |
| Space in the feeding place per head equal to or greater than 68 linear cm and easy access | |
| Space in the feeding place per head equal to or greater than 68 linear cm with anti-suffocation lifesaving system | |

Check element **40** **Size of drinking troughs (lactating cows)**

**There must be at least one drinking bowl every 10 cows or 6-7 cm/head in case of water troughs**

| | |
|---|---|
| Number or size less than the minimum required | |
| Number and size equal to the minimum required | |
| Number and size higher than expected and with well diversified locations | |

Check element **51** **Harmful gases**

| | |
|---|---|
| $NH_3$ >20 ppm; $CO_2$ >3000 ppm; $H_2S$ >0.5 ppm | |
| $NH_3$ between 10 and 20 ppm; $CO_2$ between 1500 and 3000 ppm; $H_2S$ <0.5 ppm | |
| $NH_3$ <10 ppm; $CO_2$ <1500 ppm; $H_2S$ <0.5 ppm | |

Check element **53** **Flight from humans test (lactating cows)**

**The final score is obtained by calculating the average of the scores given to each animal, rounding off to the nearest whole number**

| | |
|---|---|
| Difficulty approaching and presence of stereotypies | |
| Curious animals that can be approached and do not show stereotypies | |
| Curious animals that can be approached and let themselves be touched | |

Check element **56** **State of nutrition (lactating cows)**

**BCS values lower than 2 and greater than 4.25 are beyond acceptable limits**

| | |
|---|---|
| >10% of the animals with BCS beyond set limits | |
| <10% of the animals with BCS beyond set limits | |
| <5% of the animals with BCS beyond set limits | |

Check element **59** **Cleanliness of animals (lactating cows)**

**For each animal consider the observation of only one side**

| | |
|---|---|
| Over 20% of the animals are dirty | |
| Between 10% and 20% of the animals are dirty only in some groups | |
| Less than 10% of the animals are dirty in all the groups | |

Check element **62** **Skin lesions (lactating cows)**

**Lesions are assessed by observing the cows starting from the head and moving toward the tail: sternum, shoulder, forelimb, pelvis, thigh, hind limb (lateral and medial) and udder**

| | |
|---|---|
| Over 30% of the animals have minor skin lesions on hocks, tuberosities and soft tissues | |
| Between 15% and 30% of the animals have minor skin lesions on hocks, tuberosities and soft tissues | |
| Less than 15% of the animals have minor skin lesions on hocks, tuberosities and soft tissues | |

Check element **87** **Noise**

| | |
|---|---|
| Excessive noise | |
| Normal noise | |
